# Evidence for genetic causal relationships between gut microbiome, metabolites, and myasthenia gravis: a bidirectional Mendelian randomization study

**DOI:** 10.3389/fimmu.2023.1279845

**Published:** 2023-12-21

**Authors:** Dandan Sheng, Song Wang, Peihong Li, Jiaxin Li, Zheng Xiao, Hui Lv, Weiping Liu, Bo Xiao, Luo Zhou

**Affiliations:** ^1^ Department of Neurology, Xiangya Hospital, Central South University, Changsha, Hunan, China; ^2^ National Clinical Medical Research Center for Geriatric Diseases (Xiangya Hospital), Central South University, Changsha, Hunan, China; ^3^ Department of Pathology, First Hospital of Changsha, Changsha, Hunan, China

**Keywords:** gut microbiome, myasthenia gravis, metabolites, Mendelian randomization, causal effect, neuroimmunology

## Abstract

**Background:**

Myasthenia gravis (MG) is an autoimmune disease observed to have connections with gut microbiome. We aimed to systematically assess the causal relationships between gut microbiome, gut microbiome-derived metabolites, and MG using Mendelian randomization (MR) approach.

**Methods:**

Summary-level genetic datasets from large-scale genome-wide association studies regarding 196 gut microbial taxa from the MiBioGen consortium (n=18,340), 72 derived metabolites from the TwinsUK and KORA studies (n=7,824), and antiacetylcholine receptor (AChR) antibody-positive MG (case=1,873, control=36,370) were employed for MR causal estimates. The inverse-variance weighted (IVW) method was utilized as the main analysis with MR-Egger, maximum likelihood, simple mode, and weighted median as complements. The tests of Cochran’s Q, MR-Egger intercept, Steiger, MR-PRESSO and leave-one-out were implemented for sensitivity analyses.

**Results:**

The forward MR estimates of IVW revealed significant causal associations of the abundance of phylum Actinobacteria, class Gammaproteobacteria, family Defluviitaleac, family Family XIII, and family Peptococcaceae with a reduced risk of MG. Conversely, the abundance of phylum Lentisphaerae, order Mollicutes RF9, order Victivallales, and genus Faecalibacterium was causally associated with an increased risk of MG. The reversed MR analysis proved negative causal correlations between the MG and the abundance of family Peptostreptococcaceae, genus Romboutsia, and genus Subdoligranulum. Regarding the derived metabolites, the IVW estimates revealed that elevated levels of beta-hydroxyisovalerate and methionine were causally associated with a decreased risk of MG, while increased levels of choline and kynurenine were linked to an increased risk of MG. Furthermore, genetically predicted MG was associated with a decreased level of cholesterol. The results obtained from complementary MR methods were similar. These findings remained robust in all sensitivity analyses.

**Conclusion:**

Our MR findings support the causal effects of specific gut microbiome taxa and derived metabolites on AChR antibody-positive MG, and vice versa, yielding novel insights into prevention and therapy targets of MG. Future studies may be warranted for validation and pursuing the precise mechanisms.

## Introduction

Myasthenia gravis (MG) is an autoimmune disorder characterized by muscle weakness and fatigue. It is mediated by B-cells and associated with the presence of antibodies targeting the acetylcholine receptor (AChR), muscle-specific kinase (MUSK), lipoprotein-related protein 4 (LRP4), or agrin in the postsynaptic membrane at the neuromuscular junction (1). MG has a reported worldwide prevalence ranging from 40 to 180 per million people, with an annual incidence of 4 to 12 per million people (2). The overall in-hospital mortality rate in a cohort from the United States was 2.2% (3). In European studies, the incidence rates of MG ranged from 0.63 to 2.9 per 100,000 person-years, and prevalence rates varied between 11.17 and 36.1 per 100,000 person-years (4). Over the years, there has been a notable increase in the prevalence rate, which can be attributed to factors such as improved diagnostic precision and treatments, prolonged survival, and an aging population (5).

The patients of MG may experience muscle weakness in the oculomotor, limb, pharyngeal, and respiratory muscles. In severe cases, it can result in respiratory failure (6). The pathogenesis of MG depends on the target and isotype of the autoantibodies directed against components of the postsynaptic muscular membrane. In most cases, autoantibodies against the AChR are recognized as diagnostic markers and pathogenic factors. These autoantibodies lead to complement-mediated attack and an increase in the rate of AChR turnover, causing the loss of AChR from the postsynaptic membrane and impairing neuromuscular transmission (7, 8).

While antibodies against components of the neuromuscular junction have been identified, the exact pathogenesis of MG still remains unclear, despite its well-known multifactorial nature. The disease’s occurrence and development depend on a complex interplay of genetic and environmental factors. Notably, disruptions in gut microbiome (dysbiosis) have been a subject of particular interest in recent years, especially with advancements in human microbiome research. Growing evidence suggests that the host microbiome and its metabolites play a significant role in the pathogenesis of autoimmune diseases, including MG (9–12).

The gut microbiome plays a crucial role in maintaining the integrity of the intestinal epithelial tight junctions, thus regulating intestinal permeability. Disruptions in its composition or function can often result in increased intestinal permeability, heightening the risk of pathogenic infections and affecting Foxp3+CD4+Treg cells, thereby contributing to the development of MG (13–15). Furthermore, the gut microbiome produces a variety of metabolites, including short-chain fatty acids (SCFAs), which can modulate the T cell-proinflammatory Th17 cell axis for immune regulation and pathogen protection (13, 16). Lower levels of SCFAs are considered to be a contributing factor to the observed immune response in MG patients (17–19).

The connections between gut microbiome and MG have been established, yet the precise causal links remain elusive. Numerous studies have delved into the causality between gut microbiome and MG, primarily through case-control investigations (9). However, due to the impact of environmental factors, demographic differences and so on, the resulting conclusions exhibit variability, posing a challenge in ascertaining whether alterations in gut microbiome composition precede the onset of MG or vice versa. The genome-wide association study (GWAS) approach has demonstrated remarkable success in the identification of novel associations between genetic variants and traits, thereby facilitating the exploration of previously unknown biological mechanisms. Recently, Chia R et al. have undertaken a GWAS that included 1,873 individuals diagnosed with AChR antibody-positive MG and 36,370 healthy controls to pinpoint genetic loci influencing the risk of MG and contributing to disease susceptibility, bringing novel genetic evidence to the pathogenesis of MG (20).

Mendelian randomization(MR) is a robust and effective method using genetic variants of single-nucleotide polymorphisms (SNPs) to explore the causal effects between exposure and outcome (21). Based on the stochastic principles governing meiosis, SNPs undergo random assortment during zygote formation throughout gestation. Consequently, the outcomes of MR analyses remain impervious to reverse causation and confounding effects (22). Previous investigations utilizing MR approach have established causal links between the gut microbiome and various autoimmune and neurological disorders, encompassing type 1 diabetes, Graves’ disease, IgA nephropathy, Sjogren’s syndrome, ischemic stroke, Parkinson’s Disease, epilepsy, and migraine (23–30). However, the causal associations between gut microbiome, gut microbiome-derived metabolites, and MG have yet to be firmly established. Hence, we have undertaken a bidirectional MR study to systematically assess the underlying causalities.

## Methods

### Study design

**Figure 1:**
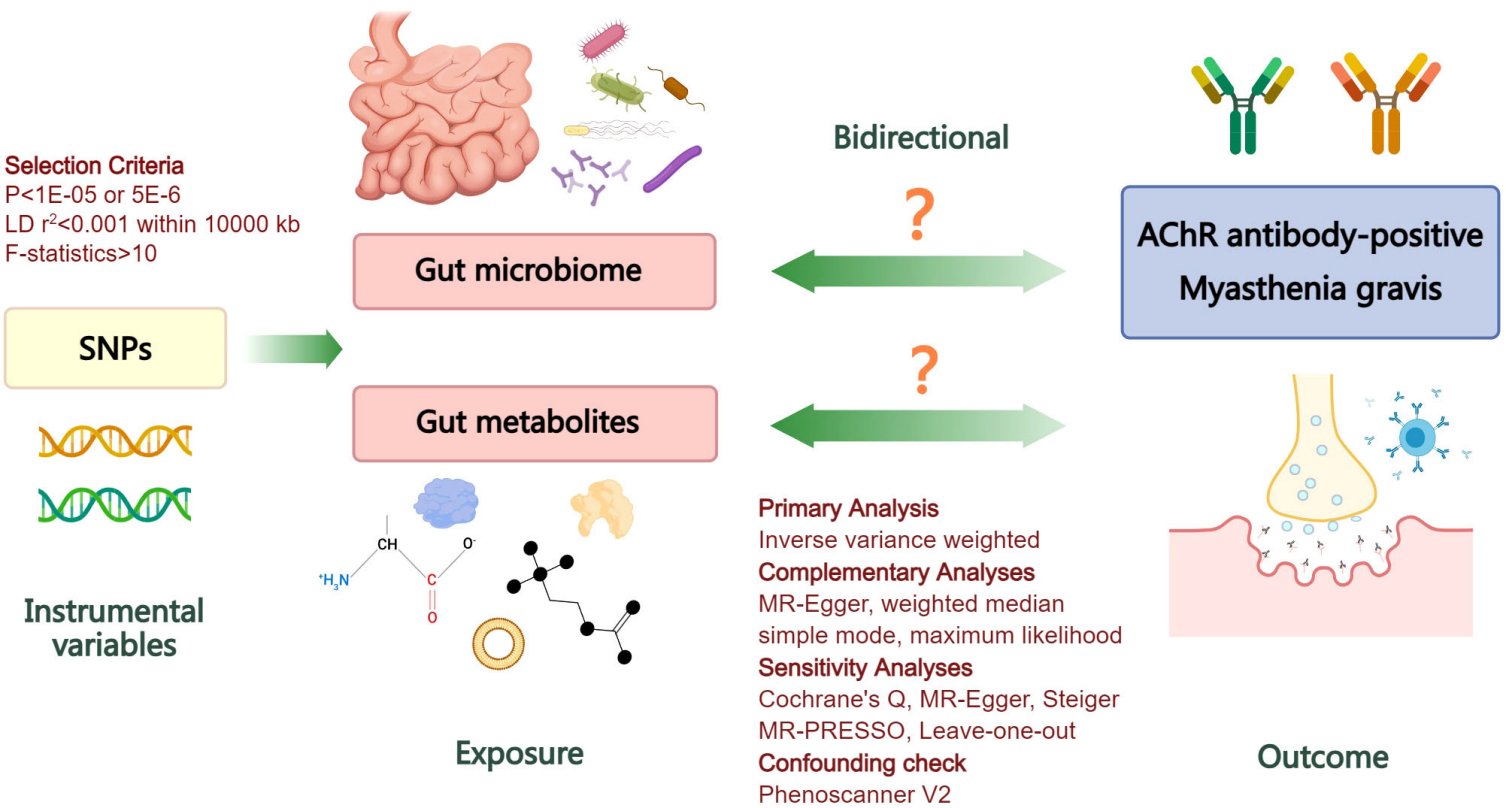
Flowchart illustrating the bidirectional MR study design for investigating the causal associations between gut microbiome, derived metabolites, and myasthenia gravis. LD, linkage disequilibrium; SNPs, single nucleotide polymorphisms; AChR, acetylcholine receptor; MR, Mendelian randomization.

Summary-level genetic datasets from large-scale GWASs of gut microbiome, gut microbiome-derived metabolites, and MG were employed for MR causal estimates. To ensure the validity of genetic variants as instrumental variables (IVs), three fundamental conditions of MR analysis must be met: firstly, the selected IVs should exhibit robust associations with the exposure; secondly, they should not be associated with any confounders of exposure or outcome; finally, the selected IVs should solely influence the outcome through their impact on exposure (21). Initially, we assessed the causal impact of gut microbiome and derived metabolites on MG in the forward analysis. Subsequently, we performed a reverse evaluation regarding the causal effect of MG on alterations in gut microbiome and derived metabolites. The flowchart of the study is depicted in **Figure 1**.

### Data source of gut microbiome

The summary-level dataset for the gut microbiome was obtained from a large-scale GWAS conducted by the MiBioGen consortium (31). This dataset, which is currently the largest genetics research of the human gut microbiome, comprises 18,340 samples collected from 24 diverse cohorts with specifical sequencing of the 16S ribosomal RNA gene. The majority of participants in these cohorts were of European ancestry. To ensure reproducibility, the microbial taxa were profiled by targeting three distinct variable regions of the 16S rRNA gene including V4 from 10,413 samples, 13 cohorts; V3-V4 from 4,211 samples, 6 cohorts; and V1-V2 from 3,716 samples, 5 cohorts. All gut microbial taxa datasets were rarefied to a standardized read count of 10,000 reads per sample. The data encompassed 211 taxa with mean abundance over 1%, including 131 genera, 35 families, 20 orders, 16 classes, and 9 phyla. Covariates such as sex, age, technical variables, and principal components were adjusted in all cohorts. For more accuracy, a total of 196 microbial taxa were included in our MR analyses, with the exclusion of 15 unidentified taxa (12 genera and 3 families) (**Supplementary Table S1**). More detailed information about this GWAS can be found in the original literature (31). The GWAS data could be obtained at https://mibiogen.gcc.rug.nl.

### Data source of gut microbiome-derived metabolites

Considering the crucial roles of metabolites in microbiome-host communication, we further employed summary-level data from a GWAS that investigated the human blood metabolome among 7,824 European participants from the TwinsUK and KORA cohorts in our current investigation (32). We conducted a manual screening for gut microbiome-derived metabolites utilizing the Human Metabolome Database (HMDB, https://hmdb.ca/). Overall, our MR analyses incorporated a comprehensive list of 72 derived metabolite traits identified through HMDB (**Supplementary Table S2**).

### Data source of myasthenia gravis

The dataset for MG was obtained from the largest recently published GWAS (20), which was conducted in cohorts from the United States and Italy, and subsequently replicated in an independent cohort from the United Kingdom. The diagnosis of MG was based on standard clinical criteria of characteristic fluctuating weakness and electrophysiological and/or pharmacological abnormalities. The presence of AChR antibodies was confirmed in all enrolled cases, as it can be detected in about 90% of patients with generalized MG. For more accuracy, patients positive for MUSK antibodies were excluded. This GWAS included a total of 1,873 cases and 36,370 controls. Among the cases, there were 989 males and 884 females with MG, while the control cohorts were well matched to the case cohorts based on ethnic group. More details of demographic profiles could be found in the original GWAS. The dataset for MG provided access to 23,679,120 SNPs, which could be obtained from IEU OpenGWAS project at https://gwas.mrcieu.ac.uk/.

### Instrumental variables selection

SNPs genetically predisposed to gut microbiome, derived metabolites, and MG were selected as IVs. The IVs had to meet specific criteria, including being free of linkage disequilibrium (pairwise r2<0.001 within 10000 kb), using the European population as a reference. We used a threshold of P<1E-05 for IV selection of gut microbiome and derived metabolites, and P<5E-06 for IV selection of MG to increase the number of SNPs available for sensitivity analyses (30). SNPs with a minor allele frequency less than 0.01 were excluded due to potentially low confidence. In order to mitigate the impact of weak genetic instruments, all SNPs selected as IVs analyzed in this study had a minimum F-statistic of 10. The directions of effects of SNPs on exposures and outcomes were then harmonized, with the exception of palindromic SNPs with intermediate allele frequencies. To minimize the correlation between SNPs and confounders, the PhenoScanner was utilized to check all IVs and the SNPs associated with potential confounding factors were removed (The detailed information of all IVs is listed in **Supplementary Tables S3-S9**).

### Mendelian randomization analyses and statistics

For the forward MR analysis, the gut microbiome and derived metabolites were chosen as the exposure traits for IVs selection with MG being considered as the outcome. Conversely, reverse MR analysis utilizing SNPs correlated with MG was performed to examine whether MG had causal impacts on gut microbial taxa and derived metabolites. The inverse-variance weighted (IVW) method, widely recognized as the most robust approach in MR studies for generating reliable causal estimates, was employed as the primary analytical tool to estimate the odds ratio (OR) and P-value in both forward and reverse directions of MR analyses. Additionally, complementary methods such as MR-Egger, maximum likelihood, simple mode, and weighted median were utilized (33). All statistical analyses were performed using the TwoSampleMR package (Version 0.5.7) in the R studio (Version 2023.06.0). Considering the exploratory nature of the study, we adopted not to perform Bonferroni correction. The significance threshold was set at a level of P<0.05.

### Sensitivity analyses

To test the robustness of the MR analyses in both forward and reverse directions. Cochran’s Q and MR-Egger intercept tests were employed to assess the presence of heterogeneity and pleiotropy among the IVs. The Steiger test was utilized to validate the accurate directions of SNPs. The Mendelian randomization pleiotropy residual sum and outlier (MR-PRESSO) in conjunction with leave-one-out analysis, were performed to identify potential outliers among the IVs. If any SNP outliers were detected, they were removed and the MR and sensitivity analyses would be re-executed (34).

## Results

### Causal effects of the gut microbiome on myasthenia gravis

The IVW estimates in forward MR analysis revealed significant causal associations between specific microbial taxa and a diminished risk of MG. These associations encompassed the phylum Actinobacteria (OR=0.60, 95% CI: 0.40-0.90, P=0.012), class Gammaproteobacteria (OR=0.59, 95% CI: 0.36-0.97, P=0.037), family Defluviitaleaceae (OR=0.70, 95% CI: 0.49-1.00, P=0.047), family Family XIII (OR=0.61, 95% CI: 0.41-0.92, P=0.017), and family Peptococcaceae (OR=0.70, 95% CI: 0.51-0.96, P=0.029). The application of the maximum likelihood method yielded consistent results across all five microbial taxa. Specifically, the phylum Actinobacteria (OR=0.61, 95% CI: 0.40-0.91, P=0.016), class Gammaproteobacteria (OR=0.58, 95% CI: 0.35-0.98, P=0.040), family Defluviitaleaceae (OR=0.69, 95% CI: 0.49-0.96, P=0.028), family Family XIII (OR=0.61, 95% CI: 0.41-0.92, P=0.018), and family Peptococcaceae (OR=0.69, 95% CI: 0.49-0.97, P=0.034) exhibited analogous causal associations with MG as IVW. Additionally, the weighted median method provided evidence supporting a causal association between Family XIII and MG (OR=0.54, 95% CI: 0.32-0.92, P=0.023).

**Figure 2:**
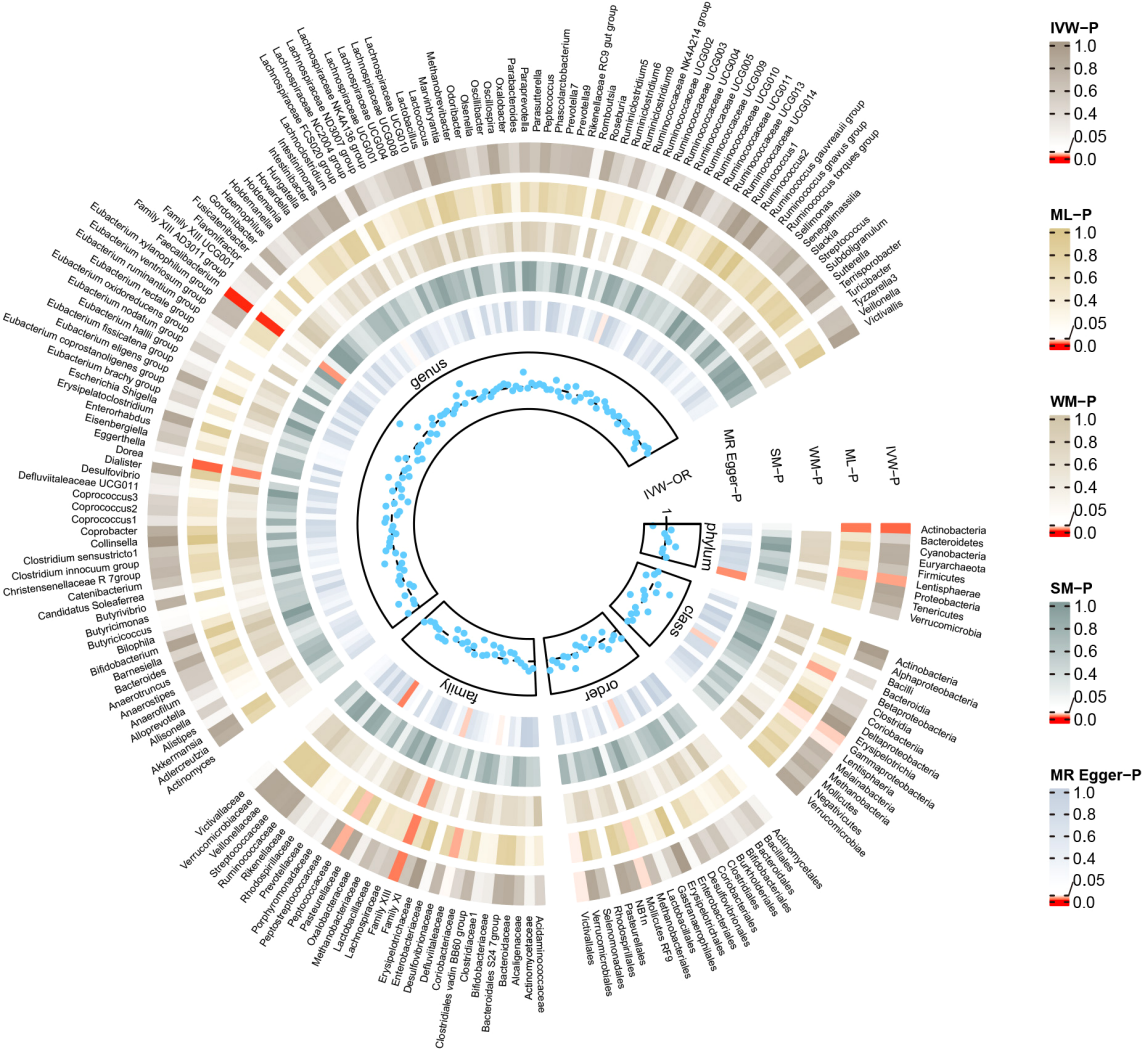
Summary of causal estimates regarding the impact of all gut microbiome on myasthenia gravis in forward MR analysis. From outside to inside, the corresponding P-values of IVW, ML, WM, SM, MR-Egger are represented, respectively. MR, mendelian randomization; IVW, inverse-variance weighted; ML, maximum likelihood; WM, weighted median; SM, simple mode; OR, odds ratio.

**Figure 3:**
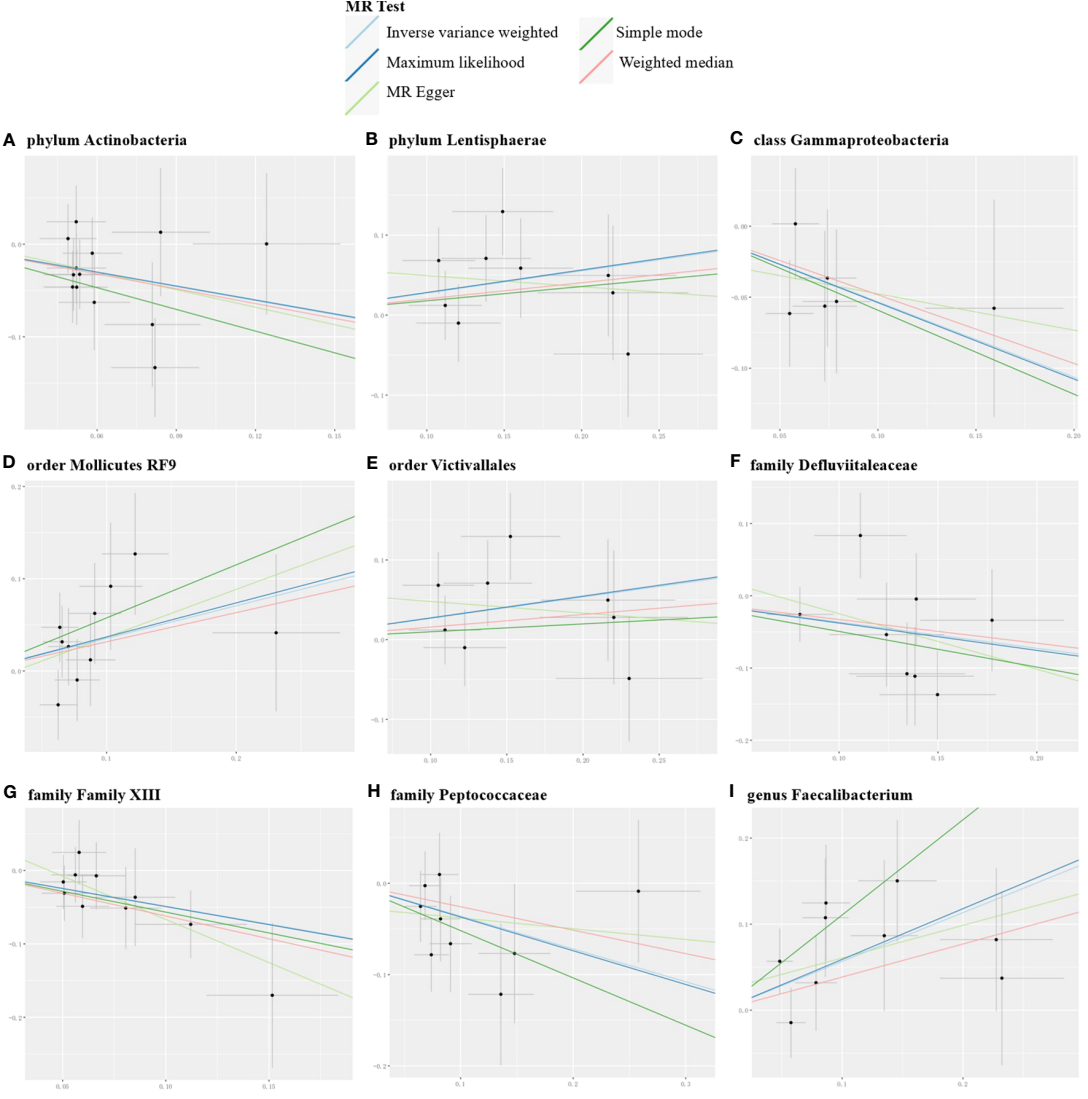
Scatter plots for the significant causal effects of gut microbiome (abscissas) on myasthenia gravis (ordinate) in forward MR analysis **(A–I)**. The presence of a positive slope signifies a positive causal association, and vice versa.

Conversely, certain microbial taxa, including the phylum Lentisphaerae (OR=1.32, 95% CI: 1.03-1.68, P=0.026), order Mollicutes RF9 (OR=1.42, 95% CI: 1.01-2.00, P=0.041), order Victivallales (OR=1.31, 95% CI: 1.01-1.69, P=0.044), and genus Faecalibacterium (OR=1.76, 95% CI: 1.22-2.55, P=0.003), were identified as risk-increasing factors for MG according to the IVW estimates. The maximum likelihood estimates confirmed the consistent correlations between these microbial taxa and the risk of MG, including the phylum Lentisphaerae (OR=1.33, 95% CI: 1.03-1.71, P=0.028), order Mollicutes RF9 (OR=1.45, 95% CI: 1.02-2.05, P=0.039), order Victivallales (OR=1.31, 95% CI: 1.01-1.72, P=0.045), and genus Faecalibacterium (OR=1.80, 95% CI: 1.22-2.67, P=0.003). Notably, the simple mode method also further supported an elevated risk for the genus Faecalibacterium (OR=3.02, 95% CI: 1.39-6.57, P=0.023) with MG. Significant MR results are presented in **Table 1**. The summary of causal estimates regarding the impact of all gut microbial taxa on MG is presented in **Figure 2**. Scatter plots illustrating the significant causal effects are shown in **Figure 3**.

**Table 1:** Significant MR results elucidating the causal effects exerted by gut microbiome on MG.

Exposures	Outcome	Methods	SNPs	OR	95%LCI	95%UCI	P
*phylum Actinobacteria*	MG	MR Egger	13	0.53	0.10	2.74	0.467
		Weighted median	13	0.59	0.34	1.01	0.052
		IVW	13	0.60	0.40	0.90	0.012
		Maximum likelihood	13	0.61	0.40	0.91	0.016
		Simple mode	13	0.46	0.17	1.26	0.157
*phylum Lentisphaerae*	MG	MR Egger	9	0.87	0.35	2.17	0.775
		Weighted median	9	1.22	0.87	1.73	0.251
		IVW	9	1.32	1.03	1.68	0.026
		Maximum likelihood	9	1.33	1.03	1.71	0.028
		Simple mode	9	1.20	0.67	2.13	0.561
*class Gammaproteobacteria*	MG	MR Egger	6	0.77	0.17	3.54	0.758
		Weighted median	6	0.62	0.32	1.19	0.150
		IVW	6	0.59	0.36	0.97	0.037
		Maximum likelihood	6	0.58	0.35	0.98	0.040
		Simple mode	6	0.55	0.22	1.38	0.261
*order Mollicutes RF9*	MG	MR Egger	10	1.68	0.65	4.31	0.313
		Weighted median	10	1.37	0.86	2.18	0.183
		IVW	10	1.42	1.01	2.00	0.041
		Maximum likelihood	10	1.45	1.02	2.05	0.039
		Simple mode	10	1.78	0.87	3.65	0.151
*order Victivallales*	MG	MR Egger	8	0.86	0.34	2.18	0.768
		Weighted median	8	1.17	0.82	1.67	0.384
		IVW	8	1.31	1.01	1.69	0.044
		Maximum likelihood	8	1.31	1.01	1.72	0.045
		Simple mode	8	1.10	0.61	1.99	0.752
*family Defluviitaleaceae*	MG	MR Egger	8	0.46	0.10	2.11	0.358
		Weighted median	8	0.72	0.47	1.11	0.139
		IVW	8	0.70	0.49	1.00	0.047
		Maximum likelihood	8	0.69	0.49	0.96	0.028
		Simple mode	8	0.61	0.30	1.23	0.210
*family Family XIII*	MG	MR Egger	10	0.31	0.09	1.09	0.106
		Weighted median	10	0.54	0.32	0.92	0.023
		IVW	10	0.61	0.41	0.92	0.017
		Maximum likelihood	10	0.61	0.41	0.92	0.018
		Simple mode	10	0.57	0.26	1.24	0.188
*family Peptococcaceae*	MG	MR Egger	9	0.89	0.41	1.91	0.771
		Weighted median	9	0.77	0.50	1.21	0.259
		IVW	9	0.70	0.51	0.96	0.029
		Maximum likelihood	9	0.69	0.49	0.97	0.034
		Simple mode	9	0.60	0.28	1.25	0.210
*genus Faecalibacterium*	MG	MR Egger	9	1.46	0.72	2.99	0.332
		Weighted median	9	1.47	0.86	2.50	0.157
		IVW	9	1.76	1.22	2.55	0.003
		Maximum likelihood	9	1.80	1.22	2.67	0.003
		Simple mode	9	3.02	1.39	6.57	0.023

SNPs, single-nucleotide polymorphisms; OR, odds ratio; LCI, low confidence interval; UCI, upper confidence interval; IVW, inverse variance weighted; MG, myasthenia gravis.

### Causal effects of the myasthenia gravis on gut microbiome

**Figure 4:**
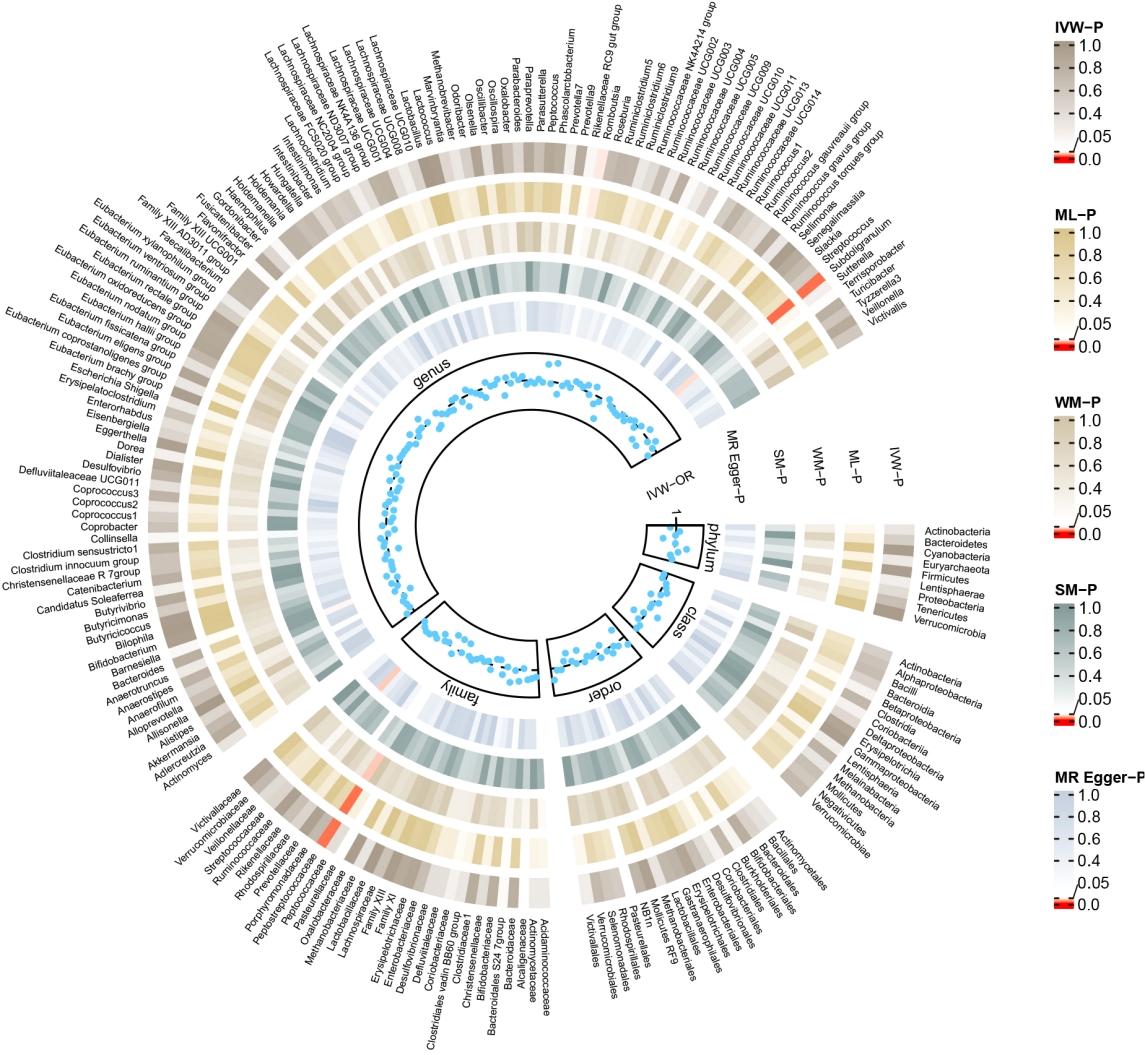
Summary of causal estimates regarding the impact of myasthenia gravis on all gut microbiome in reverse MR analysis. From outside to inside, the corresponding P-values of IVW, ML, WM, SM, MR-Egger are represented, respectively. MR, mendelian randomization; IVW, inverse-variance weighted; ML, maximum likelihood; WM, weighted median; SM, simple mode; OR, odds ratio.

**Figure 5:**
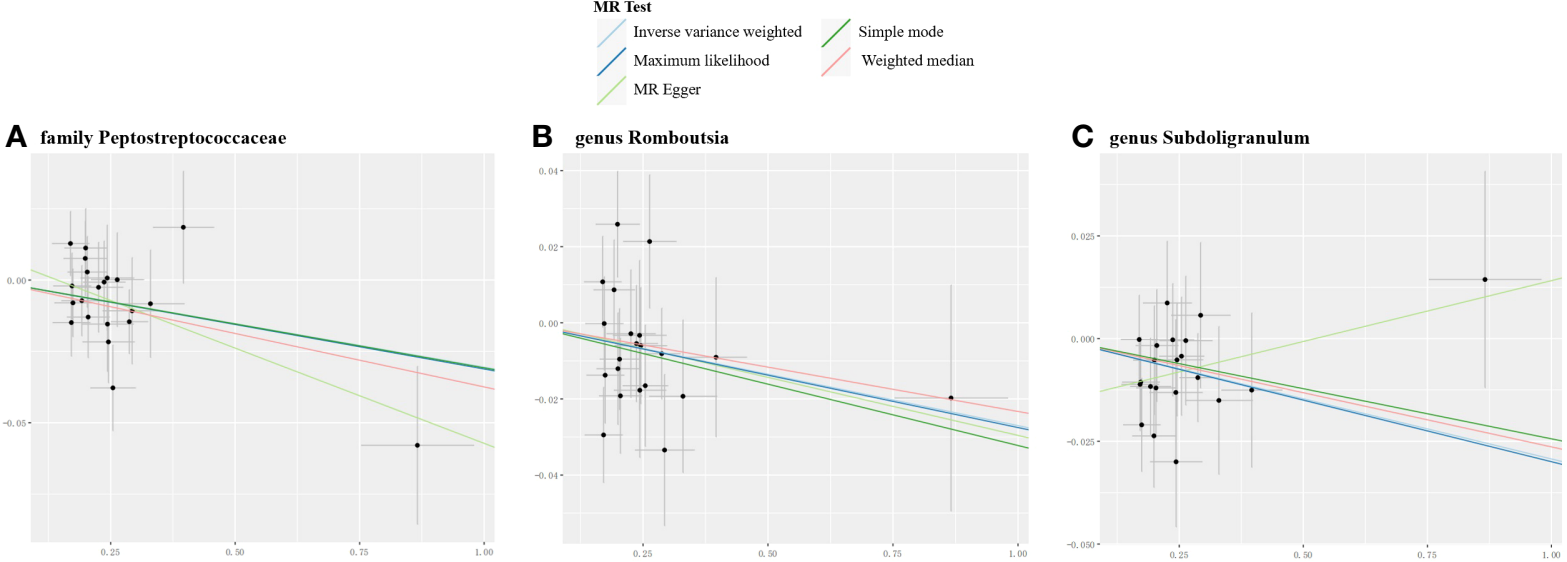
Scatter plots for the significant causal effects of myasthenia gravis (abscissas) on gut microbiome **(A–C)** (ordinate) in reverse MR analysis. The presence of a positive slope signifies a positive causal association, and vice versa.

**Figure 6:**
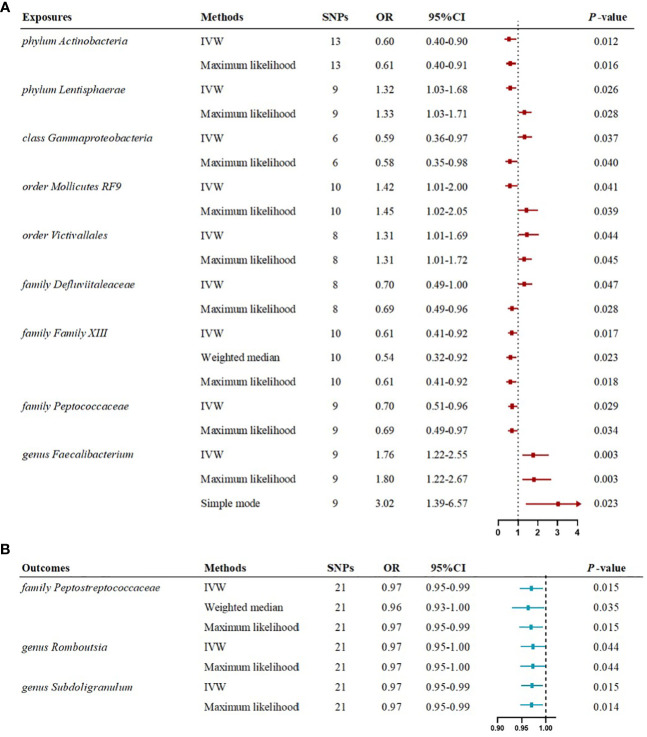
Forest plots illustrating the significant causal estimates of gut microbiome on myasthenia gravis **(A)**, and myasthenia gravis on gut microbiome **(B)** are presented. nSNPs, number of single nucleotide polymorphisms; OR, odds ratio; IVW, inverse-variance weighted.

The reverse MR analysis revealed causal effects of MG on alterations in multiple gut microbiome compositions. According to the IVW estimates, the family Peptostreptococcaceae (OR=0.97, 95%CI: 0.95-0.99, P=0.015), genus Romboutsia (OR=0.97, 95%CI: 0.95-1.00, P=0.044), and genus Subdoligranulum (OR=0.97, 95%CI: 0.95-0.99, P=0.015) exhibited inverse causal associations with the onset of MG. Maximum likelihood estimates in the family Peptostreptococcaceae (OR=0.97, 95%CI: 0.95-0.99, P=0.015), genus Romboutsia (OR=0.97, 95%CI: 0.95-1.00, P=0.044), and genus Subdoligranulum (OR=0.97, 95%CI: 0.95-0.99, P=0.014) further supported these causalities. The weighted median method also demonstrated a consistent trend with IVW in the family Peptostreptococcaceae (OR=0.96, 95%CI: 0.93-1.00, P=0.035). Significant MR results are presented in **Table 2**. The summary of causal estimates regarding the impact of MG on all gut microbial taxa is presented in **Figure 4**. Scatter plots illustrating the significant causal effects are shown in **Figure 5**. Forest plots illustrating the significant causal estimates of the five methods in bidirectional MR are presented in **Figure 6**.

**Table 2:** Significant MR results elucidating the causal effects exerted by MG on gut microbiome.

Exposure	Outcomes	Methods	SNPs	OR	95%LCI	95%UCI	P
MG	*family Peptostreptococcaceae*	MR Egger	21	0.94	0.87	1.00	0.074
		Weighted median	21	0.96	0.93	1.00	0.035
		IVW	21	0.97	0.95	0.99	0.015
		Maximum likelihood	21	0.97	0.95	0.99	0.015
		Simple mode	21	0.97	0.91	1.04	0.390
MG	*genus Romboutsia*	MR Egger	21	0.97	0.90	1.04	0.429
		Weighted median	21	0.98	0.94	1.01	0.224
		IVW	21	0.97	0.95	1.00	0.044
		Maximum likelihood	21	0.97	0.95	1.00	0.044
		Simple mode	21	0.97	0.92	1.02	0.272
MG	*genus Subdoligranulum*	MR Egger	21	1.03	0.96	1.10	0.389
		Weighted median	21	0.97	0.94	1.01	0.112
		IVW	21	0.97	0.95	0.99	0.015
		Maximum likelihood	21	0.97	0.95	0.99	0.014
		Simple mode	21	0.98	0.92	1.04	0.429

SNPs, single-nucleotide polymorphisms; OR, odds ratio; LCI, low confidence interval; UCI, upper confidence interval; IVW, inverse variance weighted; MG, myasthenia gravis.

### Causal effects of gut microbiome-derived metabolites on myasthenia gravis

**Figure 7:**
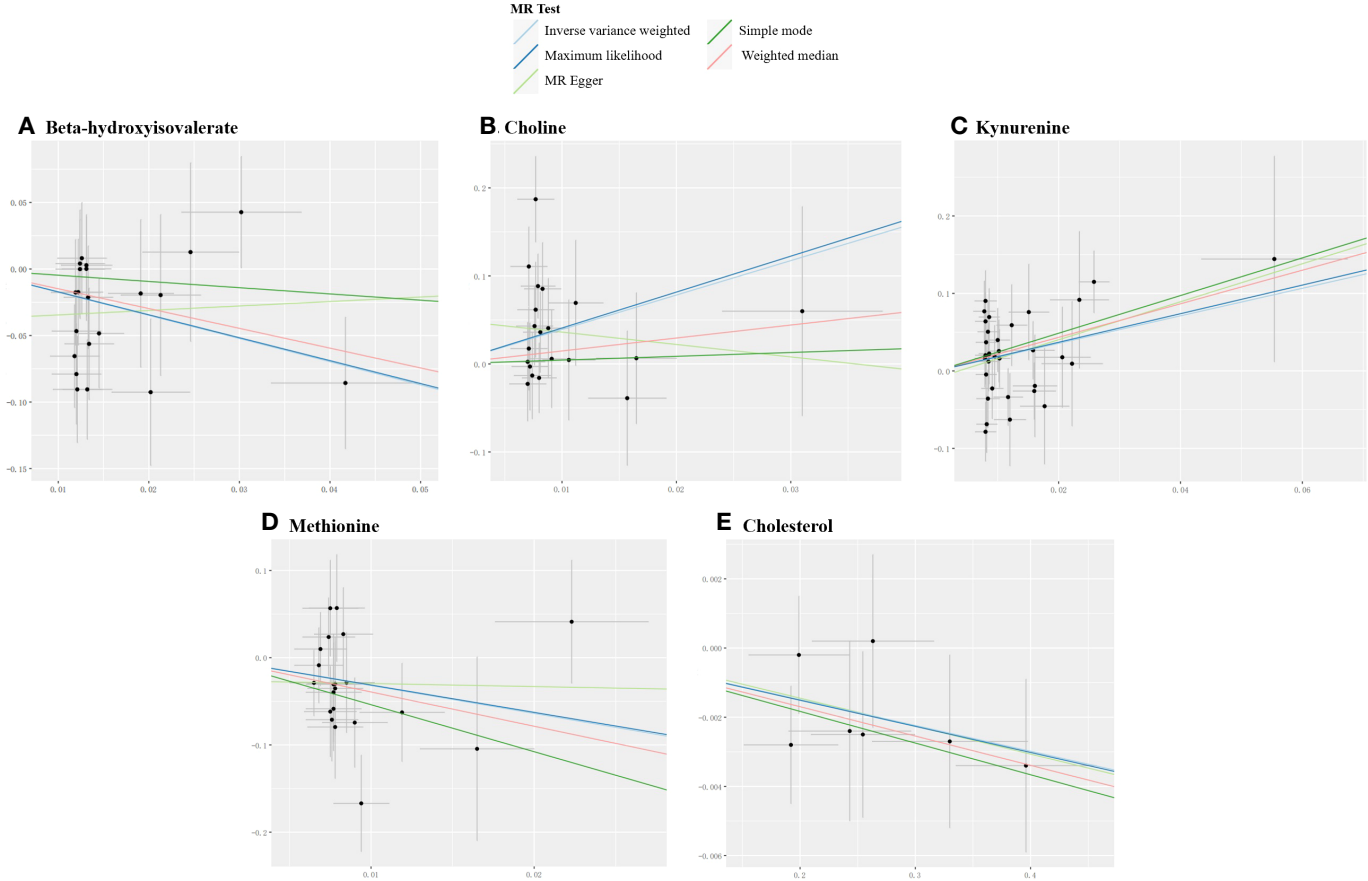
Scatter plots for the significant causal effects between gut microbiome-derived metabolites and myasthenia gravis. **(A–D)** depict the forward MR analysis, with metabolites as the exposures (abscissas) and myasthenia gravis as the outcome (ordinate). **(E)** presents the reverse MR analysis, with myasthenia gravis as the exposure (abscissas) and cholesterol as the outcome (ordinate). The presence of a positive slope signifies a positive causal association, and vice versa.

For derived metabolites, the IVW estimates in forward MR analysis revealed that the levels of beta-hydroxyisovalerate (OR=0.18, 95%CI: 0.06-0.52, P=0.002) and methionine (OR=0.04, 95%CI: 0.00-0.53, P=0.014) were inversely correlated with the risk of MG, while choline (OR=50.01, 95%CI: 2.82-885.73, P=0.008) and kynurenine (OR=5.91, 95%CI: 1.55-22.62, P=0.009) were positively correlated with the risk of MG. Consistent results were obtained using the maximum likelihood method: beta-hydroxyisovalerate (OR=0.18, 95%CI: 0.06-0.57, P=0.003), methionine (OR=0.04, 95%CI: 0.00-0.63, P=0.022), choline (OR=59.20, 95%CI: 4.33-808.53, P=0.002), and kynurenine (OR=6.33, 95%CI: 1.80-22.27, P=0.004). Weighted median estimates also indicated negative correlations for methionine (OR=0.02, 95%CI: 0.00-0.86, P=0.041) and a positive correlation for kynurenine (OR=8.72, 95%CI: 1.38-55.32, P=0.022) with the risk of MG. Significant MR results are presented in **Table 3**. Scatter plots illustrating the aforementioned significant causal effects are shown in **Figures 7A–D**.

**Table 3:** Significant MR results elucidating the causal effects between gut-derived metabolites and MG.

Exposures	Outcomes	Methods	SNPs	OR	95%LCI	95%UCI	P
*Beta-hydroxyisovalerate*	MG	MR Egger	21	1.40	0.09	20.83	0.810
		Weighted median	21	0.23	0.04	1.23	0.085
		IVW	21	0.18	0.06	0.52	0.002
		Maximum likelihood	21	0.18	0.06	0.57	0.003
		Simple mode	21	0.63	0.03	14.39	0.773
*Choline*	MG	MR Egger	20	0.24	0.00	1459.56	0.754
		Weighted median	20	4.36	0.13	150.99	0.416
		IVW	20	50.01	2.82	885.73	0.008
		Maximum likelihood	20	59.20	4.33	808.53	0.002
		Simple mode	20	1.54	0.01	380.89	0.879
*Kynurenine*	MG	MR Egger	33	11.61	0.45	301.80	0.150
		Weighted median	33	8.72	1.38	55.32	0.022
		IVW	33	5.91	1.55	22.62	0.009
		Maximum likelihood	33	6.33	1.80	22.27	0.004
		Simple mode	33	11.37	0.33	395.57	0.189
*Methionine*	MG	MR Egger	21	0.71	0.00	2806.75	0.936
		Weighted median	21	0.02	0.00	0.86	0.041
		IVW	21	0.04	0.00	0.53	0.014
		Maximum likelihood	21	0.04	0.00	0.63	0.022
		Simple mode	21	0.00	0.00	4.95	0.146
MG	*Cholesterol*	MR Egger	7	0.99	0.97	1.02	0.543
		Weighted median	7	0.99	0.98	1.00	0.029
		IVW	7	0.99	0.99	1.00	0.018
		Maximum likelihood	7	0.99	0.99	1.00	0.019
		Simple mode	7	0.99	0.98	1.00	0.144

SNPs, single-nucleotide polymorphisms; OR, odds ratio; LCI, low confidence interval; UCI, upper confidence interval; IVW, inverse variance weighted; MG, myasthenia gravis.

### Causal effects of myasthenia gravis on gut microbiome-derived metabolites

In the reverse IVW estimates, it was observed that MG was associated with a decreased level of cholesterol (OR=0.99, 95%CI: 0.99-1.00, P=0.018). This finding was consistent when employing the maximum likelihood (OR=0.99, 95%CI: 0.99-1.00, P=0.019) and weighted median (OR=0.99, 95%CI: 0.98-1.00, P=0.029) methods. The result is presented in **Table 3** and **Figure 7E**.

### Sensitivity analyses

To conduct sensitivity analyses, we confirmed the validity of IVs and MR model assumptions using Cochran’s Q and MR-Egger tests. Both the forward and reverse analyses revealed no evidence of heterogeneity or pleiotropy among IVs. The Steiger test provided evidence supporting the correctness of SNP directions. Furthermore, both MR-PRESSO and leave-one-out analyses provided robust support for our results by detecting no potential instrumental outliers. The summary of sensitivity analyses can be found in **Supplementary Tables S10-S12**. The plots illustrating leave-one-out analysis are presented in **Supplementary Figures S1**-**S3**.

## Discussion

We have conducted a comprehensive MR investigation to unravel the bidirectional causal association between the gut microbiome, derived metabolites, and AChR antibody-positive MG. Our findings unveiled a reciprocal interplay between the gut microbiome, derived metabolites and MG aligning with prior observational studies and emphasizing the pathological and physiological dynamics between them, presenting innovative insights, and indicating distinctive interactions between specific gut microbial taxa, derived metabolites and MG.

Our results underscored notable inverse causal effects on MG for the phylum Actinobacteria, class Gammaproteobacteria, family Defluviitaleaceae, Family XIII, and Peptococcaceae, implying a protective influence. Conversely, the phylum Lentisphaerae, order Victivallales, order Mollicutes RF9, and genus Faecalibacterium exerted significant positive causal impacts on MG, signifying potential risk factors.

The study conducted by Zheng et al. (35) demonstrated a reduced relative abundance of Actinobacteria in patients with MG compared to the healthy control group. Actinobacteria, one of the four major phyla of the gut microbiome, predominantly include Bifidobacteria in the human gut. Bifidobacteria are known for their beneficial effects on maintaining gut barrier integrity, attributed to their substantial production of SCFAs (4). Reduced levels of Bifidobacteria have been linked to increased gut permeability, leading to the translocation of lipopolysaccharide into the bloodstream. This event triggers immune system activation and contributes to the sustenance of chronic inflammatory conditions (36). In the older population, probiotics containing Bifidobacteria have been shown to improve constipation and enhance cellular immune activity (37), aligning with the findings of our study. Our research results are also in line with those of Sun et al. (38), who observed that nuciferine could enhance intestinal barrier functions, regulate bile acids metabolism, and alleviate inflammation in non-alcoholic fatty liver disease patients. The proposed mechanism of nuciferine metabolism involves the contribution of Family XIII, which was closely associated with specific bile acids monomers by Spearman rank correlation analysis. Interestingly, our study revealed a negative association between Gammaproteobacteria and the risk of MG. This finding contrasts with the results of Qiu et al. (18), who identified a significant enrichment in Proteobacteria. Furthermore, our study first highlighted the protective roles of the bacterial families Defluviitaleaceae and Peptococcaceae, expanding the spectrum of gut microbiome that warrants investigation in the context of MG.

The MYBIOM study, conducted in Germany, revealed an elevated abundance of Faecalibacterium in the fecal microbiome of individuals with MG compared to controls with non-inflammatory diseases, aligning with our findings (39). Faecalibacterium is known for its capacity to generate a substantial proportion of the SCFA butyrate in the intestinal milieu (40). This particular fatty acid elicits anti-inflammatory effects by activating Treg cells and enhancing the secretion of anti-inflammatory cytokines. Noteworthy is the paradoxical higher relative abundance of Faecalibacterium in MG patients, despite the disorder’s characteristic reduction in Treg cells. This may be due to the fact that the vast majority of the MG population ingests corticosteroid medications, which may affect the gut microbiome. The underlying mechanisms of this observation necessitate further exploration in subsequent research endeavors. In this MR study, intriguing associations have surfaced. Specifically, the abundance of phylum Lentisphaerae, order Mollicutes RF9, and order Victivallales exhibited correlations with the risk of MG. While prior investigations have linked Mollicutes RF9 to serum lipid levels and blood glucose in stroke patients (41), there is a dearth of research exploring the direct relationship between the phylum Lentisphaerae, order Victivallales, and MG. Subsequent research endeavors should delve into elucidating the mechanistic impact of these microbial taxa on MG, providing promising avenues for future insights.

Similarly, the onset of MG led to notable changes in gut microbiome composition. Following genetically predicted MG onset, there was a significant reduction in the abundance of the family Peptostreptococcaceae, genus Romboutsia, and genus Subdoligranulum. Notably, Chriswell ME et al.’s research established an association between a specific intestinal bacterium in the genus Subdoligranulum and various immunological phenomena, including local intestinal isolated lymphoid follicle formation, T cell and rheumatoid arthritis-associated autoantibody development, as well as paw swelling characterized by IgG, IgA, and C3 deposition in mice models (42). The family Peptostreptococcaceae has correlations with various malignancies, such as non-small cell lung cancer (43), and oral squamous cell carcinoma (44). Conversely, the genus Romboutsia is associated with metabolic disorders, particularly obesity, showing a positive correlation with parameters indicative of body weight and serum lipid levels (45). These potential mechanisms are linked to bacterial metabolism, such as producing SCFAs like butyrate and propionate, thereby fostering Treg development and functionality.

We have taken a step further to explore the causal associations between gut microbiome-derived metabolites and MG. The findings revealed that genetically predicted beta-hydroxyisovalerate and methionine demonstrated significant inverse causal effects on MG, suggesting protective factors. Conversely, choline and kynurenine exerted notable causal influences on MG, representing risk factors. A randomized controlled trial revealed that diets rich in phytochemicals and dietary fiber could enhance the abundance of beta-hydroxyisovalerate in stool samples from colorectal cancer survivors, positively modulating the patients’ gut metabolome (46). This modulation may play a significant role in MG as well. Methionine-choline deficiency can lead to abnormal mitochondrial β-oxidation function, a decrease in very low-density lipoprotein synthesis, exacerbation of inflammation, and promotion of early liver fibrosis (47). However, our MR study found that choline, which contradicts the previous consensus in nonalcoholic steatohepatitis, represented a risk factor for MG. A recent literature demonstrated that elevated concentrations of choline and the resulting increased plasma levels of trimethylamine N-oxide have been proven to be associated with heightened abundance of trimethylamine-producing bacteria and the development of cardiovascular and neurological diseases (48). Moreover, our study aligns with existing literature on the kynurenine pathway of tryptophan degradation, which was implicated in various neuropathological features presented in amyotrophic lateral sclerosis, including neuroinflammation, excitotoxicity, oxidative stress, immune system activation, and dysregulation of energy metabolism (49). Our MR findings further revealed a potentially lower level of cholesterol associated with the genetically predicted onset of MG, which may be explained by the alterations in the gut microbiome of MG individuals, potentially linked to impaired intestinal lipid absorption and dysregulated cholesterol metabolism. Of note, the metabolites analyzed in our MR study included 2-aminobutyrate, a derivative of SCFA, and 3-hydroxybutyrate, a typical SCFA, although both of which exhibited no causal association with MG.

The disparities between the outcomes of the aforementioned studies and those of our MR study might potentially be attributed to the synergistic interplay between the gut microbiome and gene-environment factors. Firstly, the use of non-selective immunosuppressive agents such as glucocorticoid in MG patients might lead to changes in the gut microbiome. Secondly, the composition of gut microbiome could vary between active and remission phases of MG, which many studies failed to consider when grouping patients. Thirdly, previous clinical observational studies were not exclusively limited to AChR antibody-positive MG; they may have also included other antibody-positive cases, such as MUSK, which could introduce confounding factors. Fourthly, the interactions and cross-talks among gut microbiome may be intricate and cannot be eliminated in former observational studies. Furthermore, factors like gender ratios, age disparities, ethnicity, and varying sample sizes across different studies could result in differences in the composition of gut microbiome. The presence of these uncertainties contributes to incongruent research outcomes and hampers the ability to infer a precise causal link between gut microbiome and MG risk. Future randomized controlled trials and pathophysiological studies are needed to provide more evidence for their interactive causal relationships.

Our study has notable strengths. Firstly, we presented the first MR study on gut microbiome, derived metabolites, and AChR antibody-positive MG. Secondly, we incorporated the latest extensive GWAS summary data, utilizing genetic information from a substantial sample size encompassing the largest cohort of MG cases. This enhances the reliability of our findings compared to small scale randomized controlled trials. To ensure robustness, bidirectional MR analyses were conducted. Furthermore, the strict inclusion criteria for the study population have made the results more convincing.

It is important to note that our study had some limitations: Firstly, the MR study did not ascertain whether there was data overlap within the included GWAS summary data. However, we have taken measures to minimize participant overlap deviation and discarding weak IVs through the utilization of F statistics (F>10). Secondly, the relatively low 10,000 read count of gene sequencing in terms of gut microbiome may be indicative of bias inherent in the original GWAS; if it was conducted using more advanced shotgun metagenomic sequencing analysis, the results would likely be more specific and accurate. Thirdly, because the majority of participants were of European ancestry, there is a possibility that the findings of this study may not be applicable to citizens in other regions. Therefore, future analyses should be conducted on a larger scale and employ more advanced methodologies to assess the interrelationships between gut microbiome features such as species and MG using high-resolution data, which may shed more light on supplementation of probiotics or prebiotics, fecal microbiota transplantation and other manners of gut microbiome regulation for MG prevention and treatment.

## Conclusions

In conclusion, our MR findings support the genetically predicted causal effects of specific gut microbiome taxa and derived metabolites on AChR antibody-positive MG, and vice versa, yielding novel insights into prevention and therapy targets of MG. Future studies may be warranted for validation and pursuing the precise mechanisms.

## Data availability statement

The original contributions presented in the study are included in the article/**Supplementary Material**. Further inquiries can be directed to the corresponding author.

## Ethics statement

This MR study did not necessitate ethical approval, as it was conducted using summarized data obtained exclusively from publicly accessible sources. No individual’s personal information was involved at any stage of this study.

## Author contributions

DS: Methodology, Software, Writing – original draft, Writing – review & editing. SW: Methodology, Visualization, Writing – original draft, Writing – review & editing. PL: Investigation, Methodology, Writing – review & editing. JL, ZX, and HL: Software, Writing – review & editing. WL: Validation, Writing – review & editing. BX: Conceptualization, Writing – review & editing. LZ: Conceptualization, Project administration, Writing – original draft, Writing – review & editing.

## References

[1] Gilhus NE Verschuuren JJ. Myasthenia gravis: subgroup classification and therapeutic strategies. Lancet Neurol (2015) 14(10):1023–36. doi: 10.1016/S1474-4422(15)00145-3 26376969

[2] Carr AS Cardwell CR McCarron PO McConville J. A Systematic Review of Population Based Epidemiological Studies in Myasthenia Gravis. *Carr et al* . BMC Neurol (2010) 10:46. doi: 10.1186/1471-2377-10-46 20565885 PMC2905354

[3] Alshekhlee A Miles MJD Katirji PB Preston DC Kaminski HJ. Incidence and mortality rates of myasthenia gravis and myasthenic crisis in us hospitals. Neurology (2009) 72:1548–54. doi: 10.1212/WNL.0b013e3181a41211 19414721

[4] Binda C Lopetuso LR Rizzatti G Gibiino G Cennamo V Gasbarrini A. Actinobacteria: A relevant minority for the maintenance of gut homeostasis. Digestive Liver Dis (2018) 50(5):421–8. doi: 10.1016/j.dld.2018.02.012 29567414

[5] Punga AR Maddison P Heckmann JM Guptill JT Evoli A. Epidemiology, diagnostics, and biomarkers of autoimmune neuromuscular junction disorders. Lancet Neurol (2022) 21(2):176–88. doi: 10.1016/S1474-4422(21)00297-0 35065040

[6] Wong SH Petrie A Plant GT. Ocular myasthenia gravis: toward a risk of generalization score and sample size calculation for a randomized controlled trial of disease modification. J Neuro-Ophthalmology (2016) 36(3):252–8. doi: 10.1097/wno.0000000000000350 27031125

[7] Verschuuren JJGM Huijbers MG Plomp JJ Niks EH Molenaar PC Martinez-Martinez P. Pathophysiology of myasthenia gravis with antibodies to the acetylcholine receptor, muscle-specific kinase and low-density lipoprotein receptor-related protein 4. Autoimmun Rev (2013) 12(9):918–23. doi: 10.1016/j.autrev.2013.03.001 23535160

[8] Gilhus NE. Myasthenia and the neuromuscular junction. Curr Opin Neurol (2012) 25(5):523–9. doi: 10.1097/WCO.0b013e3283572588 22892950

[9] Thye AY Law JW Tan LT Thurairajasingam S Chan K-G Letchumanan V. Exploring the gut microbiome in myasthenia gravis. Nutrients (2022) 14(8):1647. doi: 10.3390/nu14081647 35458209 PMC9027283

[10] Liu L Wang H Chen X Xie P. Gut microbiota: A new insight into neurological diseases. Chin Med J (Engl) (2023) 136(11):1261–77. doi: 10.1097/CM9.0000000000002212 PMC10309523 35830286

[11] Kang Y Li L Kang X Zhao Y Cai Y. Gut microbiota and metabolites in myasthenia gravis: early diagnostic biomarkers and therapeutic strategies. Clin Immunol (2022) 245:109173. doi: 10.1016/j.clim.2022.109173 36351517

[12] Kapoor B Gulati M Gupta R Singla RK. Microbiota dysbiosis and myasthenia gravis: do all roads lead to rome? Autoimmun Rev (2023) 22(5):103313. doi: 10.1016/j.autrev.2023.103313 36918089

[13] Jandhyala SM Talukdar R Subramanyam C Vuyyuru H Sasikala M Nageshwar Reddy D. Role of the normal gut microbiota. World J Gastroenterol (2015) 21(29):8787–803. doi: 10.3748/wjg.v21.i29.8787 PMC4528021 26269668

[14] Aricha R Mizrachi K Fuchs S Souroujon MC. Blocking of il-6 suppresses experimental autoimmune myasthenia gravis. J Autoimmun (2011) 36(2):135–41. doi: 10.1016/j.jaut.2010.12.001 21193288

[15] Mu L Sun B Kong Q Wang J Wang G Zhang S. Disequilibrium of T helper type 1, 2 and 17 cells and regulatory T cells during the development of experimental autoimmune myasthenia gravis. Immunology (2009) 128(1 Suppl):e826–36. doi: 10.1111/j.1365-2567.2009.03089.x PMC2753914 19740344

[16] Vijay A Valdes AM. Role of the gut microbiome in chronic diseases: A narrative review. Eur J Clin Nutr (2022) 76(4):489–501. doi: 10.1038/s41430-021-00991-6 34584224 PMC8477631

[17] Liu P Jiang Y Gu S Xue Y Yang H Li Y. Metagenome-wide association study of gut microbiome revealed potential microbial marker set for diagnosis of pediatric myasthenia gravis. BMC Med (2021) 19(1):159. doi: 10.1186/s12916-021-02034-0 34233671 PMC8265136

[18] Qiu D Xia Z Jiao X Deng J Zhang L Li J. Altered gut microbiota in myasthenia gravis. Front Microbiol (2018) 9:2627. doi: 10.3389/fmicb.2018.02627 30483222 PMC6241162

[19] Moris G Arboleya S Mancabelli L Milani C Ventura M de Los Reyes-Gavilán CG. Fecal microbiota profile in a group of myasthenia gravis patients. Sci Rep (2018) 8(1):14384. doi: 10.1038/s41598-018-32700-y 30258104 PMC6158187

[20] Chia R Saez-Atienzar S Murphy N Chiò A Blauwendraat C Roda RH. Identification of genetic risk loci and prioritization of genes and pathways for myasthenia gravis: A genome-wide association study. Proc Natl Acad Sci U.S.A. (2022) 119(5):e2108672119. doi: 10.1073/pnas.2108672119 35074870 PMC8812681

[21] Emdin CA Khera AV Kathiresan S. Mendelian randomization. JAMA (2017) 318(19):1925–6. doi: 10.1001/jama.2017.17219 29164242

[22] Sekula P Del Greco MF Pattaro C Kottgen A. Mendelian randomization as an approach to assess causality using observational data. J Am Soc Nephrol (2016) 27(11):3253–65. doi: 10.1681/ASN.2016010098 PMC5084898 27486138

[23] Luo M Sun M Wang T Zhang S Song X Liu X. Gut microbiota and type 1 diabetes: A two-sample bidirectional mendelian randomization study. Front Cell Infect Microbiol (2023) 13:1163898. doi: 10.3389/fcimb.2023.1163898 37313342 PMC10258312

[24] Cao J Wang N Luo Y Ma C Chen Z Chenzhao C. A cause–effect relationship between graves’ Disease and the gut microbiome contributes to the thyroid–gut axis: A bidirectional two-sample mendelian randomization study. Front Immunol (2023) 14:977587. doi: 10.3389/fimmu.2023.977587 36865531 PMC9974146

[25] Ren F Jin Q Liu T Ren X Zhan Y. Causal effects between gut microbiota and iga nephropathy: A bidirectional mendelian randomization study. Front Cell Infect Microbiol (2023) 13:1171517. doi: 10.3389/fcimb.2023.1171517 37201114 PMC10185820

[26] Cao Y Lu H Xu W Zhong M. Gut microbiota and sjogren’s syndrome: A two-sample mendelian randomization study. Front Immunol (2023) 14:1187906. doi: 10.3389/fimmu.2023.1187906 37383227 PMC10299808

[27] Meng C Deng P Miao R Tang H Li Y Wang J. Gut microbiome and risk of ischaemic stroke: A comprehensive mendelian randomization study. Eur J Prev Cardiol (2023) 30(7):613–20. doi: 10.1093/eurjpc/zwad052 36799937

[28] Jiang L Li JC Tang BS Guo JF. Associations between gut microbiota and parkinson disease: A bidirectional mendelian randomization analysis. Eur J Neurol (2023) 30(11):3471–7. doi: 10.1111/ene.15848 37159496

[29] Zeng Y Cao S Yang H. Roles of gut microbiome in epilepsy risk: A mendelian randomization study. Front Microbiol (2023) 14:1115014. doi: 10.3389/fmicb.2023.1115014 36922970 PMC10010438

[30] He Q Wang W Xiong Y Tao C Ma L Ma J. A causal effects of gut microbiota in the development of migraine. J Headache Pain (2023) 24(1):90. doi: 10.1186/s10194-023-01609-x 37460956 PMC10353251

[31] Kurilshikov A Medina-Gomez C Bacigalupe R Radjabzadeh D Wang J Demirkan A. Large-scale association analyses identify host factors influencing human gut microbiome composition. Nat Genet (2021) 53(2):156–65. doi: 10.1038/s41588-020-00763-1 PMC8515199 33462485

[32] Shin S-Y Fauman EB Petersen A-K Krumsiek J Santos R Huang J. An atlas of genetic influences on human blood metabolites. Nat Genet (2014) 46(6):543–50. doi: 10.1038/ng.2982 PMC4064254 24816252

[33] Yu K Chen XF Guo J Wang S Huang X-T Guo Y. Assessment of bidirectional relationships between brain imaging-derived phenotypes and stroke: A mendelian randomization study. BMC Med (2023) 21(1):271. doi: 10.1186/s12916-023-02982-9 37491271 PMC10369749

[34] Verbanck M Chen CY Neale B Do R. Detection of widespread horizontal pleiotropy in causal relationships inferred from mendelian randomization between complex traits and diseases. Nat Genet (2018) 50(5):693–8. doi: 10.1038/s41588-018-0099-7 PMC6083837 29686387

[35] Zheng P Li Y Wu J Zhang H Huang Y Tan X. Perturbed microbial ecology in myasthenia gravis: evidence from the gut microbiome and fecal metabolome. Adv Sci (Weinh) (2019) 6(18):1901441. doi: 10.1002/advs.201901441 31559142 PMC6755540

[36] Hiippala K Jouhten H Ronkainen A Hartikainen A Kainulainen V Jalanka J. The potential of gut commensals in reinforcing intestinal barrier function and alleviating inflammation. Nutrients (2018) 10(8):988. doi: 10.3390/nu10080988 30060606 PMC6116138

[37] Miller LE Lehtoranta L Lehtinen MJ. The effect of bifidobacterium animalis ssp. Lactis hn019 on cellular immune function in healthy elderly subjects: systematic review and meta-analysis. Nutrients (2017) 9(3):191. doi: 10.3390/nu9030191 28245559 PMC5372854

[38] Sun J Fan J Li T Yan X Jiang Y. Nuciferine protects against high-fat diet-induced hepatic steatosis via modulation of gut microbiota and bile acid metabolism in rats. J Agric Food Chem (2022) 70(38):12014–28. doi: 10.1021/acs.jafc.2c04817 36106619

[39] Totzeck A Ramakrishnan E Schlag M Stolte B Kizina K Bolz S. Gut bacterial microbiota in patients with myasthenia gravis: results from the mybiom study. Ther Adv Neurol Disord (2021) 14:17562864211035657. doi: 10.1177/17562864211035657 34394728 PMC8361534

[40] Qiu X Zhang M Yang X Hong N Yu C. Faecalibacterium prausnitzii upregulates regulatory T cells and anti-inflammatory cytokines in treating tnbs-induced colitis. J Crohns Colitis (2013) 7(11):e558–e68. doi: 10.1016/j.crohns.2013.04.002 23643066

[41] Li N Wang X Sun C Wu X Lu M Si Y. Change of intestinal microbiota in cerebral ischemic stroke patients. BMC Microbiol (2019) 19(1):191. doi: 10.1186/s12866-019-1552-1 31426765 PMC6700817

[42] Chriswell ME Lefferts AR Clay MR Hsu AR Seifert J Feser ML. Clonal iga and igg autoantibodies from individuals at risk for rheumatoid arthritis identify an arthritogenic strain of subdoligranulum. Sci Transl Med (2022) 14(668):eabn5166. doi: 10.1126/scitranslmed.abn5166 36288282 PMC9804515

[43] Vernocchi P Gili T Conte F Del Chierico F Conta G Miccheli A. Network analysis of gut microbiome and metabolome to discover microbiota-linked biomarkers in patients affected by non-small cell lung cancer. Int J Mol Sci (2020) 21(22):8730. doi: 10.3390/ijms21228730 33227982 PMC7699235

[44] Zhang L Liu Y Zheng HJ Zhang CP. The oral microbiota may have influence on oral cancer. Front Cell Infect Microbiol (2019) 9:476. doi: 10.3389/fcimb.2019.00476 32010645 PMC6974454

[45] Zeng Q Li D He Y Li Y Yang Z Zhao X. Discrepant gut microbiota markers for the classification of obesity-related metabolic abnormalities. Sci Rep (2019) 9(1):13424. doi: 10.1038/s41598-019-49462-w 31530820 PMC6748942

[46] Brown DG Borresen EC Brown RJ Ryan EP. Heat-stabilised rice bran consumption by colorectal cancer survivors modulates stool metabolite profiles and metabolic networks: A randomised controlled trial. Br J Nutr (2017) 117(9):1244–56. doi: 10.1017/S0007114517001106 PMC5654571 28643618

[47] Wang Q Zhou H Bu Q Wei S Li L Zhou J. Role of xbp1 in regulating the progression of non-alcoholic steatohepatitis. J Hepatol (2022) 77(2):312–25. doi: 10.1016/j.jhep.2022.02.031 35292349

[48] Hosseinkhani F Heinken A Thiele I Lindenburg PW Harms AC Hankemeier T. The contribution of gut bacterial metabolites in the human immune signaling pathway of non-communicable diseases. Gut Microbes (2021) 13(1):1–22. doi: 10.1080/19490976.2021.1882927 PMC7899087 33590776

[49] Ning J Huang S-Y Chen S-D Zhang Y-R Huang Y-Y Yu J-T. Investigating casual associations among gut microbiota, metabolites, and neurodegenerative diseases: A mendelian randomization study. J Alzheimer’s Disease: JAD (2022) 87(1):211–22. doi: 10.3233/JAD-215411 35275534

