# Supplementary figures and images for: Evidence for genetic causal relationships between gut microbiome, metabolites, and myasthenia gravis: a bidirectional Mendelian randomization study
Source: Front Immunol. 2023 Dec 21;14:1279845. doi: 10.3389/fimmu.2023.1279845 (PMC10764630; doi:10.3389/fimmu.2023.1279845)

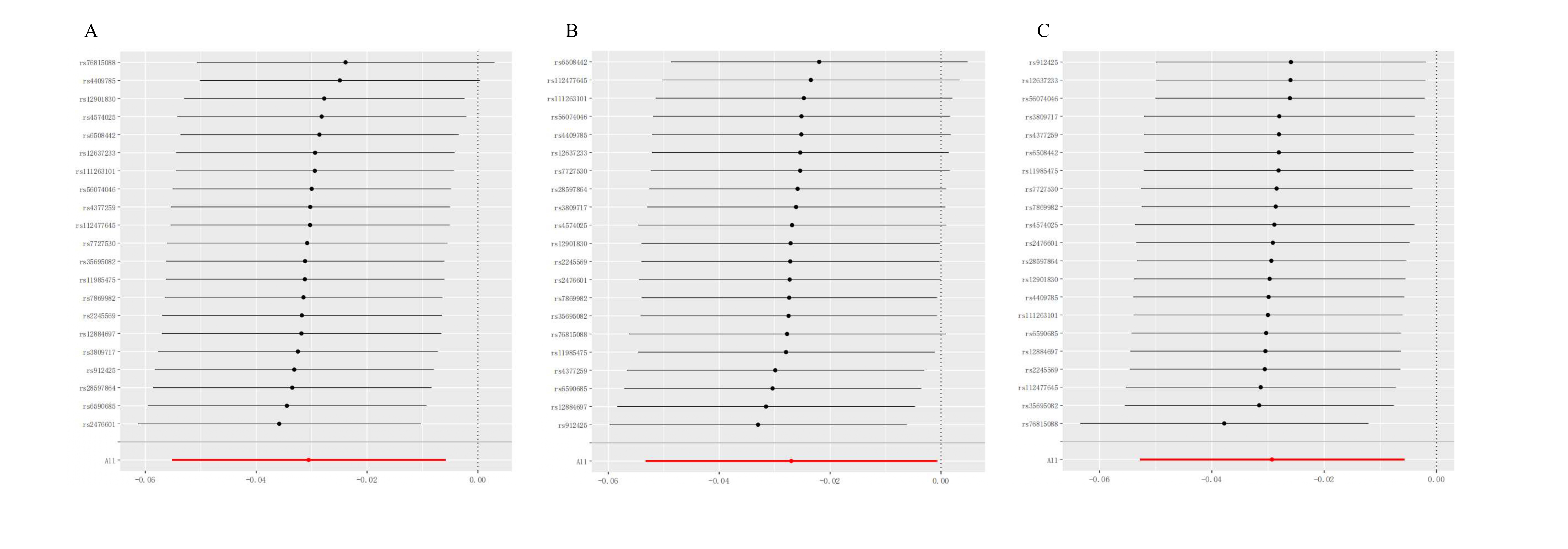

Supplement: Supplementary file 2 [file Image_2.jpeg]

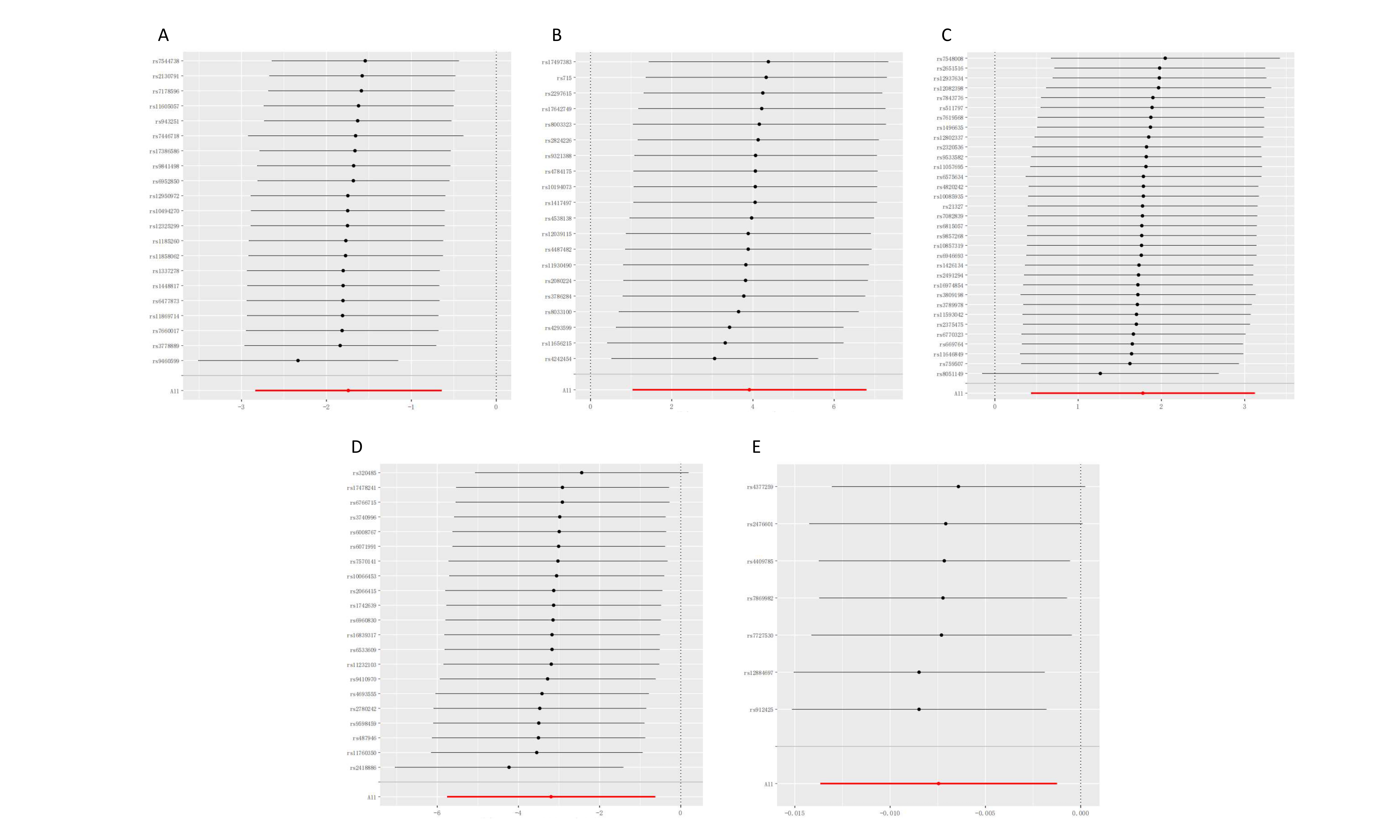

Supplement: Supplementary file 3 [file Image_3.jpeg]
